# Editorial

**Published:** 1866-10

**Authors:** 


### Editorial.

Clinical Instruction in Chicago.—When the writer came to this city, in the autumn of 1849, there was nothing bearing the name of a hospital in it; and the clinical instruction was limited to one or two college clinics per week, during the annual lecture term of sixteen weeks. The following summer, he aided in the procurement of *twelve* beds, in a building rented temporarily, for the beginning of a public hospital, and in the autumn of 1850, commenced the work of hospital clinical instruction. This constituted the beginning of what has since been known as Mercy Hospital, and which has been increased until it now holds permanent possession of one of the finest buildings and locations in the city. It is constantly well filled with patients, under the immediate care of the Sisters of Mercy, with the professors of Practical Medicine and Surgery in the Chicago Medical College as its medical and surgical attendants. Its wards are open for clinical instruction four days in the week, during nine months of each year, *viz.:*—from the first Monday in October, to the first Monday in July. The full clinical course in this hospital constitutes an essential part of the curriculum of the Chicago Medical College.

In addition to this, Chicago now affords clinical instruction, two days in each week, in the United States Marine Hospital, by Dr. R. N. Isham, Prof. of Surgical Anatomy and Operations of Surgery, in the Chicago Medical College; two days in each week, in the Cook Co. Almshouse Hospital, by Dr. J. P. Ross, one of the attending physicians, and Dr. G. K. Amerman, one of the attending surgeons, and in the eye and ear ward, once a week, by Dr. Hildreth, the attending surgeon in that department; and one day in each week, in St. Luke's Hospital, by the attending surgeon, Dr. Owens. Both the colleges also maintain two clinics per week, on patients from their respective dispensaries. We have made this enumeration, simply to remind the students and profession in the North-west, that in no city East,

West, or South, can the student find better advantages for true bedside clinical instruction than here in Chicago.

---

MORTALITY FOR THE MONTH OF AUGUST.—

CAUSES OF DEATH.

| | | | |
|---|---|---|---|
| Accidents, | 11 | Fever, Typhus, | 1 |
| Bronchitis, | 1 | Fever, not stated, | 3 |
| Burned, | 3 | Fever, Childbed, | 2 |
| Cancer, | 3 | Hydrocephelus, | 6 |
| Childbed, | 5 | Inflammation of Kidneys, | 1 |
| Cholera, | 139 | Inflammation of Brain, | 16 |
| Cholera-Morbus, | 15 | Inflammation of Bowels, | 15 |
| Cholera Infantum, | 25 | Inflammation of Lungs, | 13 |
| Colic, | 1 | Inflammation of Pleura, | 1 |
| Congestion of Brain, | 5 | Killed, | 1 |
| Congestion of Lungs, | 3 | Measles, | 47 |
| Consumption, | 33 | Old Age, | 15 |
| Convulsions, | 37 | Paralysis, | 1 |
| Cramp, | 9 | Pneumonia, | 3 |
| Disease of Brain, | 3 | Rheumatism, | 2 |
| Disease of Heart, | 2 | Scrofula, | 1 |
| Disease of Liver, | 5 | Small-Pox, | 1 |
| Disease of Spine, | 2 | Stillborn, | 11 |
| Delirium Tremens, | 2 | Summer Complaint, | 217 |
| Decline, | 3 | Suicide, | 2 |
| Dropsy, | 12 | Teething, | 37 |
| Diarrhœa, | 38 | Tuberculosis, | 1 |
| Diphtheria, | 7 | Whooping-Cough, | 63 |
| Dysentery, | 29 | Worms, | 1 |
| Erysipelas, | 1 | Unknown, | 31 |
| Fever, Remittent, | 19 | | |
| Fever, Scarlet, | 17 | Total, | 940 |
| Fever, Typhoid, | 14 | | |

AGES OF THE DECEASED.—Under 5 years, 578; over 5 and under 10 years, 46; over 10 and under 20, 34; over 20 and under 30, 78; over 30 and under 40, 78; over 40 and under 50, 48; over 50 and under 60, 27; over 60 and under 70, 25; over 70 and under 80, 7; over 80 and under 90, 2; over 100, 1; unknown, 16. Total, 940.

NATIVITIES.

| | | | | | |
|---|---|---|---|---|---|
| Chicago, | 548 | France, | 4 | Sweden, | 9 |
| Other States, | 127 | Holland, | 3 | Unknown, | 2 |
| Canada, | 9 | Ireland, | 69 | | |
| Denmark, | 6 | Norway, | 28 | Total, | 940 |
| England, | 12 | Bohemia, | 10 | | |
| Germany, | 98 | Scotland, | 4 | | |

DIVISIONS.

| | | | | | |
|---|---|---|---|---|---|
| North, | 252 | South, | 297 | West, | 386 |
| County, (out of city), | 3 | Unknown, | 12 | Total, | 940 |

| | |
|---|---|
| Number of deaths in July, | 706 |
| Increase, | 234 |
| Number of deaths August, 1865, | 466 |
| Increase, | 474 |

### REPORT OF THE COMMITTEE ON THE SANITARY CONDITION OF THE CITY, AND THE PREVALENCE OF DISEASES, DURING THE MONTH OF JULY, 1866.

By N. S. DAVIS, M.D., of Chicago.

Read before the Chicago Medical Society, August, 1866.

*Atmospheric Conditions.*—During the first seven days of July, the winds were from the south and south-west; the temperature high; more or less rain every day, except the 4th and 5th; atmosphere excessively damp and deficient in electricity, inducing in the animal system a feeling of languor and oppression. The only change during these seven days, was on the evening of the 2d, when the wind suddenly changed to the north-west, bringing a cooler atmosphere during the night, but which was again reversed in the morning. On the 8th, the wind had changed to the north-east, and the atmosphere continued chilly and damp through the day. 9th, wind from the north, and, atmosphere still cold, clear, and less damp. 10th and 11th, wind south-east; atmosphere warm, but clear, dry, and pleasant. From the morning of the 12th, to the evening of the 17th, the wind was from the south and south-west; the atmosphere continuously hot, damp, and deficient in free electricity, inducing a marked feeling of oppression and weakness. About 1 o'clock P.M., of the 17th, the wind suddenly changed to the north-east, bringing a cool, damp atmosphere, and a slight sprinkle of rain; and in the evening, copious showers, with thunder and lightning. During the 18th, 19th, and 20th, the wind continued north and north-east, with a cool, clear, but somewhat damp atmosphere. During the evening of the 20th, it became cloudy, with the wind more to the south. 21st, wind south, cloudy, hot, and oppressive, with copious showers in the evening, followed by a cool north-east wind, and a fine display of atmospheric electricity. About midnight, the wind again changed to the south, and the morning of the 22d was hot, rainy and oppressive. The 23d, 24th, 25th, only moderately warm, and wind mostly east and north-east, but showers occurred every day or night, keeping the air saturated with dampness. The 26th, 27th, and 28th, the wind was almost continuously from the south; the atmosphere filled with clouds, mist, and sometimes rain, and very hot. During the night of the 28th, the wind changed to the north, and the 29th 30th, and 31st were cool, for the most part clear and pleasant.

It will thus be seen, that the most prominent meteorological characteristics of July were, high temperature; frequent rains; excessive dampness; south and south-west winds, with deficient free atmospheric electricity. The principal exceptions to this rule were from the 17th to the 21st, and from the 29th to the 31st. The average temperature of the month was here, as in most other sections of our county, above the average of the preceding ten years. I have been particular to mention the direction of the winds, because those from the south, south-west, and west come over an almost continuous rich, alluvial prairie surface, and in the summer always bring an atmosphere not only elevated in temperature, but deficient in both electricity and ozone. Its effects upon the human system are popularly expressed by the words, *relaxing*, *sultry*, *oppressive*. Those from the east, north-east, and north, sweep over an equally continuous surface of fresh, pure water, extending 40 miles east and hundreds of miles north; and in summer never fail to bring a cool, invigorating, though often damp atmosphere.

In regard to local sanitary conditions, it is proper to state that, although the scavenger system adopted by the city authorities has served to keep the streets and alleys of the central part of the city some cleaner than in former years, yet up to the last day of July there were extensive sections of the city, thickly covered with dwellings, in which not even a street gutter had been cleaned out; and both branches of the Chicago River have been in their usually offensive condition. As might have been anticipated, from the local and atmospheric conditions just detailed, the prevalence of intestinal diseases in the city, during the month of July, has been much above the average of the preceding three or four years.

In a preceding report, it was stated that from the 20th to the 27th of June there was a prevalence of south winds, with a hot, damp, and oppressive atmosphere, during which, "serous diarrhœa and dysentery increased rapidly among young children, and to some extent among adults." The cool invigorating atmosphere of the last three days of June checked this tendency in some degree, but the renewal of the hot, damp, and showery weather, during all the first seven days of July, again increased rapidly the number of attacks of cholera-morbus, cholera infantum, diarrhœa, and dysentery. During the 6th and 7th, particularly, the attacks of bowel-affections were not only numerous, but they were accompanied by unusual languor, prostration, and sweating. The days were hot, cloudy, with some rain, and slight wind from the south. Two cases of sunstroke were reported on the 7th.

On the night of the 6th and morning of the 7th, I saw three or four children presenting symptoms of great prostration; countenances pale; pulse slow and weak; respiration slow and inefficient; mental faculties dull or in a state of semi-sleep, from which it was difficult to arouse them; occasional sighing and momentary restlessness; but with little or no evacuations of any kind. Their paleness and apparent lifelessness was the chief cause of alarm to the parents and friends. I have noticed a few similar cases during the first extremely hot days and nights of almost every summer. They appear to be the result of a deficiency of atmospheric oxygen and electricity, coupled with the relaxing influence of a high temperature. During the 8th, 9th, 10th, the number of new attacks of bowel-affections was decidedly less, and most of those that did occur, presented the form of dysentery. But from the 11th to the 17th, the cases of serous diarrhœa and cholera-morbus, especially among children, increased so rapidly that two-thirds of all the patients that came under my observation during that period were of this class.

Among my daily entries in the note book, is the following: "During the night of the 16th, the air was very hot and relaxing. Both that and the preceding night, children with bowel-complaints became very restless and prostrate, with discharges thin as water. Several were taken suddenly prostrate, drowsy, and feverish, without any discharges." Other periods of rapid increase of new attacks took place during the 21st and 22d, and from the 26th to the 28th, inclusive. According to my notes, four-fifths of the cases of serous diarrhœa and cholera-morbus that occurred during the whole month, had their *commencement* either between the 1st and 7th; the 11th and 17th; 21st and 22d; or the 26th and 28th, while the cases of dysentery occurred more evenly throughout the month. If these dates are compared with the detail of atmospheric conditions, it will be seen that a hot and damp atmosphere invariably accompanied the unusually rapid multiplication of cases of serous diarrhœa and cholera-morbus. Are these atmospheric conditions, coupled with the idio-miasmatic influences generally present in densely populated cities, capable of causing attacks of genuine spasmodic cholera? As bearing on this question, I will simply relate the following facts:—

On the night of the 7th of July, one of the most oppressive that occurred during the month, a lady living at 160 Western Avenue, on the extreme western limits of the city, began to have disturbance of the bowels with serous discharges, which culminated in severe cholera symptoms on the night of the 8th, including the characteristic rice-water discharges, by vomiting and purging; severe cramps in the muscles of the extremities and abdomen; a small, weak pulse; skin shrivelled and cool; voice husky, etc. Under appropriate treatment she recovered.

CASE II. On the morning of the 10th, was called to Mr. L., at 200 Huron Street, in the North Division of the city. He was then presenting all the symptoms of the active stage of spasmodic cholera, but which had been preceded by moderate diarrhœa since the evening of the 7th.

CASE III. On the morning of the 11th, a lady from Kansas arrived at the Adams House. She had been much fatigued by the long journey, and had suffered from a moderate diarrhœa during the three preceding days. Soon after her arrival, the diarrhœa began rapidly to increase, and at noon, she was presenting every symptom of severe cholera. The discharges up and down were copious, rice-water in appearance, and accompanied by severe muscular cramps in the extremities. Two hours later, when I first saw her, the eyes were deeply sunken; the extremities cold and skin shrunken; the pulse extremely weak; the voice reduced to a whisper; the cramps severe; and the intestinal discharges wholly involuntary. Under treatment, the discharges ceased, reaction took place slowly, and the patient ultimately recovered.

Case IV. A man, aged about 20, baker by occupation, residing at 172 West Lake Street, was attacked suddenly, during the afternoon of the 12th. The cholera symptoms were strongly marked and violent in every respect. I saw him about two hours after the commencement of the attack. His system responded promptly to the action of medicine, and he recovered. The day was excessively hot, and the man had indulged in drinking eight or ten glasses of beer, and two or three of brandy, besides a considerable quantity of water.

Case V. Mr. K., aged about 55 years, long in infirm health, was attacked with violent symptoms of cholera on the night of the 6th. When I saw him, on the 7th, he was in collapse. Partial reaction took place, and he lingered until the 10th, when he died. He resided on the corner of West Indiana and Sangamon Streets.

Case VI. Mr. W., aged about 45 years, residing at 245 Madison Street, was attacked on the night of the 15th. Early in the morning of the 16th, I found him presenting all the symptoms of the active stage of genuine cholera. But he responded to the action of remedies and recovered.

Case VII. Mr. D., aged 35 years, intemperate habits, residing on Franklin Street, near Jackson, was attacked on the night of the 15th. When I saw him, early in the morning of the 16th, he appeared to be in the commencement of collapse. Reaction, however, took place; he lingered several days with symptoms of secondary fever, but finally recovered.

Cases VIII., IX., X. On the morning of the 22d, I was called in quick succession to three cases of adults, who had been suddenly attacked during the latter part of the night previous with vomiting, purging, cramps, etc. They were seen during the early part of the active stage, and readily yielded to treatment.

Such were the indications of cholera in this city during the month of July, as they came under my own observation. The cases were not reported to the Health-Officer, because they were sporadic, having no connection with each other or with any importation from other places, and because they gave no evidence of being either contagious or infectious. But if these cases originated from atmospheric and local causes, why may not a more active development of the same causes give rise to the same disease in its more malignant and epidemic form? The only difference I could detect between these sporadic cases and cases of cholera in the midst of an epidemic, consisted in less depression of vital susceptibility, and a consequent more prompt response to the action of medicines.

That the month of July was attended by a much greater proportion of bowel-affections than usual, is apparent from the very great increase of mortality during that month. The whole number of deaths for June was 319, and for July 706; the excess in the latter month being almost wholly from bowel-affections.

---

The Cholera Cases in this City During the Month of September, 1866.—The subjoined report of the Health-Officer, gives the number of deaths from cholera, in this city, during the past month, showing the number for each ward, their nativity, and ages:—

*Nativity.*—United States, 33; Bohemia, 9; Canada, 2; Denmark, 2; England, 6; France, 2; Germany, 110; Holland, 1; Ireland, 66; Norway, 19; Poland, 1; Sweden, 5; Switzerland, 2; Spain, 1; Scotland, 1; Italy, 1; unknown, 11. Total, 272.

*Wards.*—First Ward, 20; Second Ward, 21; Third Ward, 19; Fourth Ward, 16; Fifth Ward, 9; Sixth Ward, 15; Seventh Ward, 21; Eighth Ward, 8; Ninth Ward, 5; Tenth Ward, 5; Eleventh Ward, 19; Twelfth Ward, 37; Thirteenth Ward, 3; Fourteenth Ward, 35; Fifteenth Ward, 20; Sixteenth Ward, 14; unknown, 2. Total, 272.

*Ages.*—Under 5 years, 15; over 5 and under 10 years, 22; over 10 and under 20, 15; over 20 and under 30, 64; over 30 and under 40, 74; over 40 and under 50, 40; over 50 and under 60, 23; over 60 and under 70, 7; over 70 and under 80, 1. Total, 272.

Chicago Medical College.—The Eighth Annual Course of instruction in this Institution commenced on Monday evening, Oct. 1st. The exercises commenced with prayer by the Rev. Dr. Goodspeed. The general introductory lecture was given by Prof. M. O. Heydock, and was an elegantly written address. The lecture-room was well filled with an attentive and interested audience. After the lecture, the museum, library, and other rooms were thrown open for the inspection of the visitors. Every part of the edifice was in excellent order, and supplied with everything that could contribute to the convenience and progress of the student. The number of students present was much larger than at the commencement of any previous term since the college was organized.

---

Disinfection and Deodorization.—Dr. Herbert Barker, the successful competitor for the Hastings Prize Essay for 1865, was led, by a series of observations and experiments on this subject, to the following conclusions:—

1. For the sick-room, free ventilation, when it can be secured, together with an even temperature, is all that can be required.
2. For rapid deodorization and disinfection, chlorine is the most effective known.
3. For steady and continuous effect, ozone is the best agent known.
4. In the absence of ozone, iodine, exposed in the solid-form to the air, is the best.
5. For the deodorization and disinfection of fluid and semi-fluid substances undergoing decomposition, iodine is best.
6. For the deodorization and disinfection of solid bodies that cannot be destroyed, a mixture of powdered chloride of zinc, or powdered sulphate of zinc with sawdust, is best. After this, a mixture of carbolic acid and sawdust ranks next in order, and following on that, wood-ashes.
7. For the deodorization and disinfection of infected articles of clothing, etc., exposure to heat at 212° Fahr. is the only true method.
8. For the deodorization and disinfection of substances that may be destroyed, heat to destruction is the true method.—*Journal of Chemistry.*

